# Nanoscale Biosensors Based on Self-Propelled Objects

**DOI:** 10.3390/bios8030059

**Published:** 2018-06-25

**Authors:** Beatriz Jurado-Sánchez

**Affiliations:** 1 Department of Analytical Chemistry, Physical Chemistry and Chemical Engineering, University of Alcalá, E-28871 Alcala de Henares, Madrid, Spain; beatriz.jurado@uah.es; Tel.: +34-918854941; 2 Chemical Research Institute “Andrés M. del Río”, University of Alcala, E-28871 Alcala de Henares, Madrid, Spain

**Keywords:** micromotor, nanomotor, biosensing

## Abstract

This review provides a comprehensive overview of the latest developments (2016–2018 period) in the nano and micromotors field for biosensing applications. Nano and micromotor designs, functionalization, propulsion modes and transduction mechanism are described. A second important part of the review is devoted to novel in vitro and in vivo biosensing schemes. The potential and future prospect of such moving nanoscale biosensors are given in the conclusions.

## 1. Introduction

According to the IUPAC, a biosensor can be defined as “a device that uses specific biochemical reactions mediated by isolated enzymes, immunosystems, tissues, organelles or whole cells to detect chemical compounds usually by electrical, thermal or optical signals”. Since the first biosensor developed by Clark in 1962 [1], the field has been the focus of a strong research interest. The emergence of nanotechnology along with the success of the microelectronics industry have motivated the miniaturization of biosensors into the nano/microscale. Indeed, the reduction of the dimensions of the biosensing element has been shown to improve the sensitivity of the overall system by increasing the system’s signal-to-noise ratio size. Additional advantages include portability, easy-to-use-features, reduced cost and materials requirements along with the possibility to perform multiplexed analysis in the same chip, package, or system [2,3]. 

The major challenge in biosensor miniaturization is the adequate trade-off between sensor dimensions–signal transduction efficiency and reaction-transport kinetics. Strategies to increase detection performance include the separation of microelectrode systems by nanogaps or the decrease of the thickness of gate dielectric in chemical field effect transistors biosensors. Strategies to reduce the overall response time associated with mass transport limitations compromise the use of external forces such as pressure-drive flow or internal forces such as self-propelled objects to replenish the analyte-depleted solution [3]. Indeed, micro/nanomotors hold considerable potential for developing novel biosensing protocols involving ‘on-the-move’ recognition and sensing events. Such moving objects are designed to perform selected mechanical movements in response to specific stimuli. They are built from a few micro- and nanoscale components, each of which can be chemically or biologically functionalized, and operate on distinct actuation principles (local fuels or magnetic and ultrasound energies) [4,5,6,7,8,9,10]. 

The aim of this review is to provide a current overview of recent advances in the use of self-propelled micromotors in biosensing applications. As excellent reviews have comprehensively covered the use of catalytic micromotors in bio-affinity sensing and cell isolation [11,12,13,14], we will describe here the latest developments since 2016. A brief overview is summarized in Table 1, which we hope will help the reader to follow the contents of this review. In the following sections, we will discuss material design aspects and transduction mechanisms of such exciting moving machines, to conclude with recent applications for “in vitro” and “in vivo” biodetection and future prospects in the field. 

## 2. Moving Biosensor Design: Materials, Propulsion and Transduction Mechanisms

**Figure 1:**
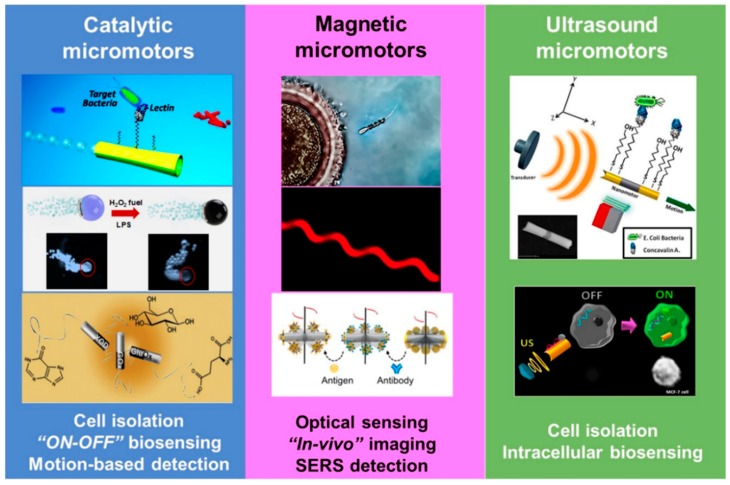
Nano and micromotors “at work” in biosensing schemes and related applications. Catalytic micromotors: illustrating lectin-modified tubular micromotors for bacterial isolation (top part), quantum dots loaded catalytic Janus micromotors for endotoxin detection based on fluorescence quenching (middle part) and motion-based detection of glucose, xanthine and glutamate based on enzyme-powered nanowires (bottom part). Magnetic micromotors: a magnetic propelled helix carrying a sperm cell to an oocyte (top part), a fluorescent microscopy image of a spirulina-based magnetite micromotors for bioimaging (middle part) and antibody modified magnetic actuated nanowires in SERS detection operations (bottom part). Ultrasound micromotors: Lectin-modified nanowires for bacterial isolation (top part) and microRNA intracellular sensing using modified nanowires. Reprinted with permission from ref. [35], American Chemical Society; ref. [25], Wiley; ref. [16], Elsevier; ref. [36], American Chemical Society; ref. [30], The American Association for the Advancement of Science; ref. [31], American Chemical Society; ref. [32] American Chemical Society and ref. [33] American Chemical Society.

The choice of a given material and the specific propulsion mechanism are critical factors influencing the application of micromotors for biosensing applications. Figure 1 shows a schematic of the most commonly used nano- and micromotors classified according to their propulsion mechanism as well as related applications. Transduction mechanisms can be either optical, electrochemical, SERS and even positron emission tomography. Most micromotors are composed of polymeric, carbon and magnetic segments (mainly for catalytic propulsion) but current trends are exploring bio-inspired designs using vascular plants, red blood cells, bacteria or sperm cells as base or functional components of the micromotor body (mainly for magnetic and ultrasound propulsion). This chapter is organized into four subsections, three devoted to the role of fabrication, functionalization and propulsion mechanisms of catalytic, magnetic and ultrasound micromotors in biosensing schemes. The last subsection is devoted to transduction mechanisms, which are common for the different types of micromotors. 

### 2.1. Catalytic Micromotors 

**Figure 2:**
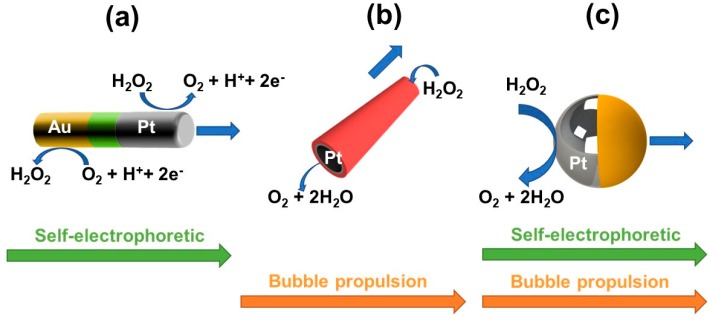
Catalytic micromotors for biosensing applications and related propulsion mechanisms. (**a**) Nanowires; (**b**) Tubular micromotors and (**c**) Janus micromotors.

Catalytic micromotors, which rely on a chemical input fuel for efficient propulsion, are the most commonly studied and widely applied for biosensing applications. Various designs have been developed depending on the specific mechanism, i.e., self-electrophoresis, self-diffusioelectrophoresis and bubble propulsion (see Figure 2). Bimetallic Au–Pt nanowires and Janus micromotors rely on self-electrophoretic propulsion mechanisms. The fuel, usually hydrogen peroxide, is oxidized to oxygen on the Pt segment while on the Au segment the hydrogen peroxide is reduced to water. Movement of the hydronium and/or other positive ions drags the liquid close to the layer via viscosity forces and creates an electroosmotic flow on the surface of the nanomotor, which consequently moves in the opposite direction. Self-diffussioforetic can also occur due to the creation of a concentration gradient across the particle interfacial region and cause water to flow from regions of low to high solute concentrations, generating a fluid flow that propels the motor. Yet, a limitation of such motors is the hampered locomotion in salt-rich media, which limits their application to motion-based detection sensing approaches [4,37,38]. Bubble-propelled micromotors, pioneered by Wang and Schmidt, display a tubular or rolled up structure with an outer polymeric or carbon nanomaterial layer and an inner catalytic layer, commonly platinum [7,39,40]. The oxygen-bubble propulsion mechanism is associated with the catalytic decomposition of the fuel at the inner catalytic layer, which produces oxygen gas that nucleates into bubbles. The conical shape promotes the unidirectional expansion of the catalytically generated oxygen bubbles, and their release from one of the tubular openings, pushing the micromotor forward [41]. Janus micromotors half-covered with a catalytic Pt layer can also decompose the peroxide fuel, generating oxygen gas bubbles responsible for the micromotor propulsion in the opposite direction. Asymmetry here is key for promoting oxygen bubbles accumulation in one size of the micromotor for directional propulsion [42]. Tubular and Janus micromotors are extremely attractive for biosensing purposes due to their rich outer surface chemistry for further functionalization but most importantly to avoid the ionic-strength limitation, allowing for their application in relevant biological media [13].

Enzymes can be also used as alternatives to catalytic metals to power nano- and micromotors either using peroxide fuel or its corresponding substrate. Early designs rely on functionalization of the inner or outer gold layers of the micromotors with the enzyme (mainly catalase). Enzyme catalysis of hydrogen peroxide as fuel induces fluid flow and bubble generation for efficient propulsion. Recent trends are aimed at enzyme coupling to the surface of Janus-like or tubular structures, with the enzymatic turnover of substrates as the energy to overcome random Brownian motion. Yet, efforts in this direction have been directed toward the search for biocompatible fuel rather than for biosensing purposes [43,44,45]. However, the dependence of the micromotor velocity on the substrate fuel concentration can be exploited for motion-based biosensing approaches, which will be described in the following sections.

Different synthetic approaches have been adopted for the synthesis of micromotors, which can exert some influence in the overall nano- and micromotors functionality for receptors immobilization or encapsulation in biosensing schemes. Nanowires are prepared by template-assisted electrodeposition using porous alumina or polycarbonate membranes as templates [46,47]. Different metallic segments are sequentially reduced/deposited within the pores of the membrane. Composition can be tailored by using different metallic solutions, allowing to add Ni segments for magnetic guidance [48] or carbon nanomaterials for enhanced speed [49]. The experimental set-up is similar to a regular electrochemical cell, with an Ag/AgCl electrode and a Pt wire as reference and counter electrodes, respectively. The membrane is transformed into a working electrode by sputtering a gold or silver thin layer. Tubular micromotors can be synthetized by template electrodeposition of an outer polymeric/carbon nanomaterials layer and an inner catalytic metal tubular layer [7,28,40,47] or by rolled-up technology. In the latter one, a photoresist is used for the sequential electron-beam electrodeposition of different metallic layers or polymeric/metallic layers. Subsequent sonication induces the roll of the as-deposited layer, resulting in a conical tube [8,29,50]. In both cases, the rich outer surface chemistry of both systems (with carbon or gold layers) allow for the incorporation of bioreceptors (antibody, lectins) and highly efficient quenching application schemes. Janus nano/micromotors are commonly prepared by asymmetric physical vaporization of a thin layer of a material (normally Pt) on spherical particles [42]. Oil-in-water emulsion approaches offer higher versatility for the encapsulation of engineering nanoparticles for biosensing and propulsion elements [25].

Once the micromotor body is synthetized, it can be easily modified to incorporate the biosensing units adapting commonly used procedures in macroscale sensors. As can be seen in Table 1, the gold layer of nanowire motors can be modified with enzymes or thiolated DNA for motion-based detections schemes. The generation of reaction products (from enzymatic degradation of substrates or the duplex formation of the nucleic acid target with the DNA tagged with silver nanoparticles that dissolve in solution) induced an increased speed of the self-diffussioforetic-propelled nanowire motors which can be tracked with an optical microscope and related to the target analyte concentration [15,16]. Antibodies, lectins or aptamers can be incorporated into the outer gold or carbon-based layer of tubular or rolled-up micromotors via 1-Ethyl-3-(3-dimethylaminopropyl)carbodiimide (EDC)/N-Hydroxysuccinimide (NHS) chemistry for cell isolation and “on-off” detection [13,51]. The high versatility of the template electrodeposition techniques can be exploited for the preparation of micromotors with ‘built-in’ recognition properties of the outer polymeric layer [21,22].

### 2.2. Magnetic Micromotors 

**Figure 3:**
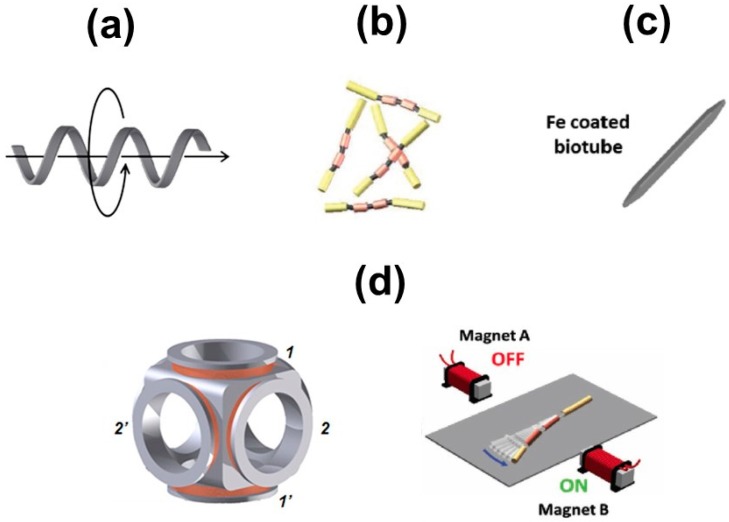
Magnetic micromotors for biosensing applications and related propulsion mechanisms. (**a**) Helical swimmers; (**b**) Flexible nanowires and (**c**) Microdraggers. Part (**d**) shows and schematic of the propulsion mechanisms based on Helmholtz coils (left part) or permanent magnets (right part). Reproduced with permission from ref. [53] (**a**,**d**), American Chemical Society; ref. [54] (**b**,**d**), Wiley and ref. [55] (**c**), Wiley.

Magnetic fields hold considerable promise to achieve fuel-free propulsion at the nano- and microscale. Since fields with low strength are not harmful for cells and tissues, magnetic micromotors open new avenues form intracellular and in vivo biosensing schemes. Magnetic fields can be either used for directional control of micromotors (tubular, nanowires) for lab-on-a-chip [52] and magneto immunoassays or as the driving force of the propulsion itself. Compared to catalytic-propelled nano- and micromotors, magnetic actuated propellers are still in their early stages. Different designs and main propulsion mechanisms are depicted in Figure 3.

Helical micromotors propel through the generation of linear thrusts from their corkscrew-like rotation in a low-Reynolds number medium. Flexible swimmers or nanowires propulsion rely on a propagating wave traveling along their flexible parts, which induce their undulatory propulsion mechanism. Plan filaments have been coated with magnetic materials and subsequently applied for cell drilling towards nanosurgery applications. In all cases, the micromotors can be propelled by means of oscillating or rotating magnetic fields, generated via Helmholtz coil or electromagnets (see Figure 3d). Magnetic micromotors have been manufactured by direct laser writing, vapor deposition on inorganic and organic templates or template assisted electrodeposition routes. Excellent reviews have covered in detail the propulsion mechanism and fabrication [56,57].

To date, few reports in the literature have described the use of magnetic micromotors in biosensing schemes. Yet, the magnetic body can be easily modified with receptors such as antibodies for immunoassays in connection with SERS detection [31]. Of particular interest, spirulina have been used as biotemplate for the preparation of magnetic helices, with the native bacteria fluorescent as the sensing element in bioimaging [30]. Helical micromotors can be directly used for whole cell capture (i.e., sperm cells) for optical detection [36]. More details will be described in the following section.

### 2.3. Ultrasounds Micromotors 

**Figure 4:**
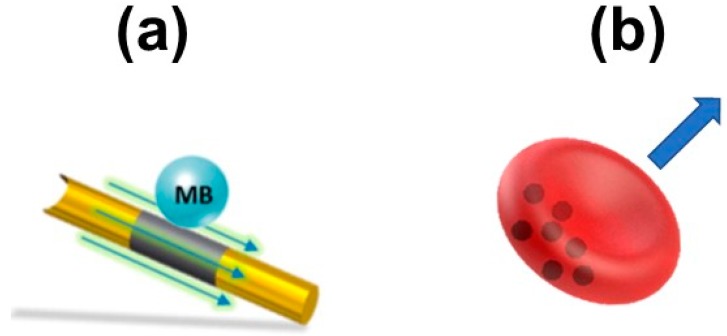

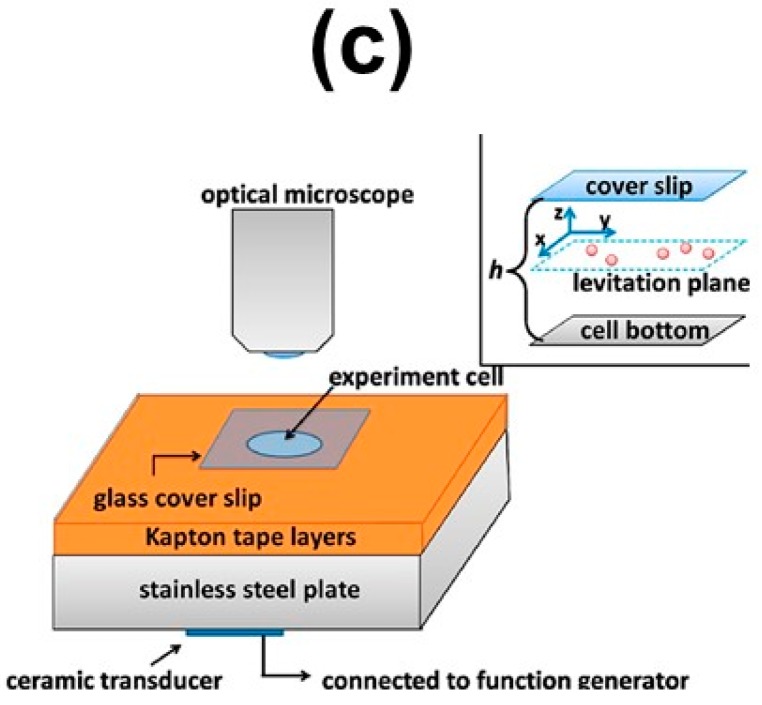
Ultrasound micromotors for biosensing applications and related propulsion mechanism. (**a**) Nanowires; (**b**) Iron-oxide loaded red blood cells (**c**) Schematic of the experimental set-up and propulsion mechanism. Reproduced with permission from ref. [32] (**a**), American Chemical Society; ref. [58] (**b**), American Chemical Society and [59] (**c**), American Chemical Society.

Ultrasound is another type of biocompatible source which can be employed in intracellular detection schemes. Yet, it is somewhat more complex than catalytic and magnetic propulsion and nanomotor design is key for getting the best propulsion performance. For sensing purposes, nanowires motors with a concave end [32] or red-blood cell motors [58] with asymmetric distribution of iron oxide nanoparticles (see Figure 4) are used. The propulsion mechanism is based on self-acoustophoresis, resulting from the asymmetry of the nanomotors which had one convex and one concave end. The local pressure gradient formed in the concave end push the micromotors forward. More details and additional propulsion/control mechanism and additional configurations, which are out of the scope of this review, can be found in the literature [59,60]. 

For biosensing purposes, the gold segment of ultrasound-propelled nanowires can be easily functionalized with antibody, lectins of aptamers for cell isolation and RNA detection. Red-blood cells can be easily loaded with nanoparticles such as fluorescent quantum dots for bioimaging and theragnostic applications. More details are described in following sections.

### 2.4. Transduction Mechanisms 

**Figure 5:**
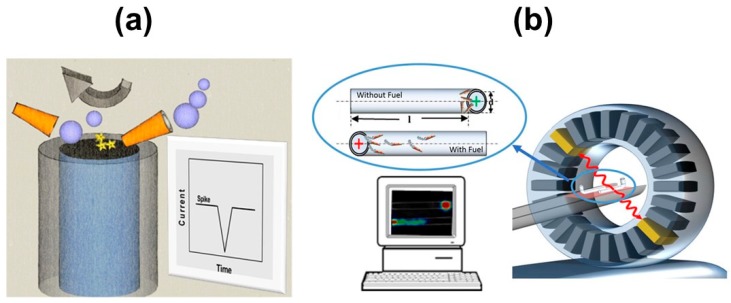
Transduction mechanisms for micromotors tracking in biosensing schemes. (**a**) Electrode impact voltammetry: schematic of a micromotor impacting a carbon microfiber electrode surface and the corresponding electrochemical signal generated. (**b**) Positron emission tomography. Reproduced with permission from ref. [61] (**a**), American Chemical Society and ref. [62] (**b**), American Chemical Society.

Typically, biosensors employ optical, electrochemical, mass-sensitive or thermal transduction mechanisms. The nanosized dimensions of self-propelled micromotors and their moving nature demand a new design for proper detection. Motion-based and optical detection can be performed using high-performance optical microscopes, which remains a challenge for future point-of-care integration and decentralized analysis. Efforts in this direction have been aimed to achieve visual colorimetric detection or electrochemical approaches by previous isolation of the micromotor in a chip reservoir, with only the analytes of interest (previously captured by the micromotors) being transported to a glassy carbon electrode for detection [24]. Another convenient approach introduced by Pumera relies on particle-electrode impact voltammetry for real-time evaluation of micromotor motion, opening new avenues for the development of novel mobile sensors during micromotor operation in environmental and biological media (see Figure 5a) [61]. For in vivo detection, Sanchez’s group have reported the use of positron emission tomography, which is widely used in medical imaging, to track iodine isotope-labelled tubular micromotors, opening new avenues for future applications (see Figure 5b) [62].

## 3. In Vitro Biosensing 

Catalytic micromotors are uniquely suited for in vitro biosensing approaches due to their superior enhancing mixing effect and high towing force to accelerate biosensing approaches without compromising sensitivity and overall performance. Most importantly, such moving biosensors can be used directly in unprocessed biological samples, yielding high recoveries and reducing the overall analysis time. Yet, the requirements for toxic peroxide fuel prevent its application in “in vivo” detection schemes [13,63].

**Figure 6:**
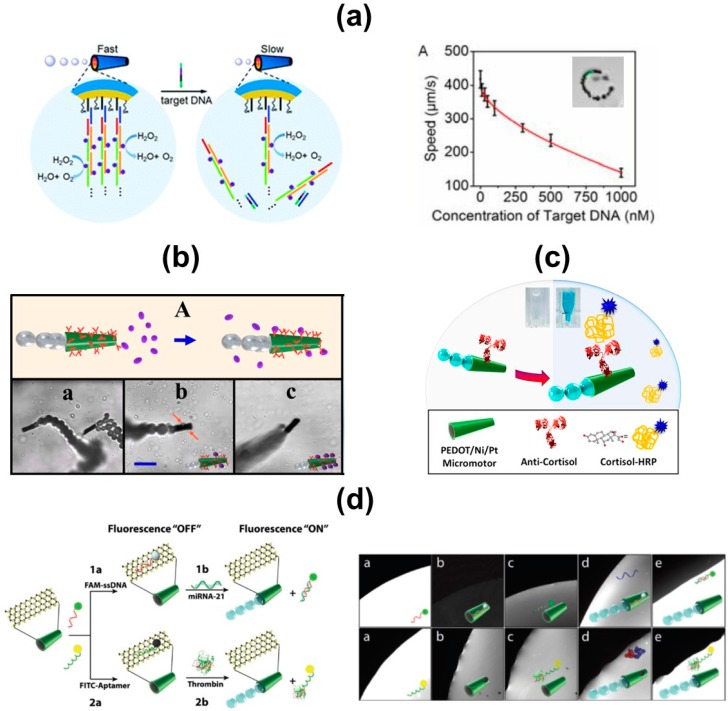
In vitro biosensing using self-propelled micromotors. (**a**) Motion-based DNA detection using PEDOT-catalase-DNA micromotors and graph showing the dependence on the speed upon DNA concentration. (**b**) Antibody-modified PEDOT micromotors for *Bacilus globigii* spore isolation and optical detection. (**c**) Antibody-modified PEDOT micromotors for colorimetric detection of cortisol. (**d**) MoS_2_ micromotors for “on-off” detection of microRNA and proteins. Reproduced with permission from ref. [18] (**a**), Royal Society of Chemistry; ref. [65] (**b**), Royal Society of Chemistry; ref. [23] (**c**), Elsevier and ref. [28] (**d**), Wiley.

We will describe here the progress in the field from early motion-based detection approaches to cutting edge “on-off” detection schemes (see Table 1 and Figure 6). Initial attempts rely on nanowire structures (Au–Pt) which display enhanced motion in the presence of silver ions. Such an effect was attributed to the underpotential deposition of silver onto the Pt segment, which increases the electrocatalytic activity. Changes in the speed of the nanowires can be tracked using an optical microscope [64]. The strategy was then extended for DNA detection, using a silver-tagged detection probe which hybridizes selectively with the target DNA [15]. Gold nanowires integrating a PPy segment and modified with the enzymes glucose oxidase, glutamate oxidase or xanthine oxidase display specific acceleration in the presence of its corresponding substrates in a concentration-dependent manner [16]. Recent motion-based approaches are translating such initial efforts to tubular configurations to avoid the hampered locomotion in salt-rich environments. Thus, “signal on” DNA biosensors based on PEDOT/Au micromotors were introduced as alternatives. One configuration relies on platinum nanoparticles-DNA conjugates as catalysts to propel the micromotors in a hydrogen peroxide solution. The catalyst is only attached to the motor in the presence of the DNA target (turn-on characteristics) [17]. A second configuration based on enzymatic propulsion is depicted in Figure 6a. The micromotor body was fabricated by sequential assembly of multiple catalase layers on the inner Au surface of the PEDOT microtube. Catalase was immobilized using a designed sandwich DNA structure as the sensing unit, and then alternately hybridizing with two assisted DNAs to binding the enzyme for efficient motor motion. In the presence of target DNA, the sensing unit hybridized with the target DNA, releasing the catalase (essential for propulsion) which resulted in the decrease of the motion speed (see Figure 6 right part) [18].

Tremendous research efforts in the field have also been aimed at the functionalization of tubular structures for whole cell isolation and visual detection. Rolled-up micro-engines with an outer gold layer have been functionalized with anti-carcinoembryonic antigen for cancer cell isolation [19]. Electrochemical detection of HL-60 leukemia cells involves the use of aptamer-modified PEDOT/Ni micromotors as preconcentration/transport units. After capturing cells from a human serum sample, the micromotors were separated by magnetic forces and used to transport the cancer cells to a clean microchip chamber. Next, releasing aptamer was added to release the HL-60 cells, which are determined by electrochemical impedance spectroscopy. Simultaneously, the micromotors were directed to another reservoir for further reuse [24]. The described micromotor approach is relevant in the medical field and for its application in real samples. Even if the ratio is 1 object: 1 cell, micromotor-based assays are performed using high quantities/number of such functionalized probes (10^5^–10^6^ in number) to meet the criteria for clinical diagnosis. Tubular PANI micromotors prepared by template-assisted electrodeposition and modified with an outer AuNPs layer (via layer-by-layer assembly) were modified with specific antibodies of cancer biomarkers. Such powerful microsensor allowed for in situ visualization immunoassays through motion readout or tag counting using an optical microscope [20]. Wang’s group employed polymers rich in carboxylic groups to synthetize the micromotor body. Such negative groups allow for the incorporation of specific antibodies via EDC/NHS chemistry. Figure 6b shows an example of such type of micromotors, which were functionalized with *Bacilus globigii* antibodies for the selective isolation of whole cells of the biochemical weapon [65]. Visual colorimetric detection has been achieved with PEDOT micromotors, as depicted in Figure 6c. Anti-cortisol-functionalized-micromotors were employed for the rapid isolation of an HRP tagged cortisol target. Short incubation of the resulting cortisol–HRP-modified micromotors with 3 3′ 5 5′-tetramethylbenzidine and peroxide solution result in a deep blue colored solution for rapid detection [23]. Another convenient approach relies on the synthesis of micromotors with built-in recognition, which have been used for selective yeast cell or proteins isolation avoiding the use of specific receptors or antibodies [21,22].

Recent efforts in the field have been directed to the design of fluorescent-based bioassays based on micromotors, using engineered particles such as quantum dots or dye-labelled aptamers. Graphene/Pt micromotors functionalized with a fluorescein–amidine-tagged ricin B aptamer were used for “on-off” detection of toxins in food and biological samples [27]. Our group synthetized magnetocatalytic Janus micromotors encapsulating PABA-modified graphene quantum dots as sensing units. The native micromotor fluorescence (imparted by the quantum dots) was quenched upon interaction with the target endotoxin or lipopolysaccharide, whereby the PABA tags acted as highly specific recognition receptors of the LPS core polysaccharide region. The strategy was applied for *Escherichia coli* and *Salmonella enterica* endotoxin detection in clinical and food samples [25,26]. Chalcogenides such as MoS_2_ are also promising materials for “on-off” fluorescent approaches in connection with labelled probes. Figure 6d illustrates an example of MoS_2_/Pt tubular micromotors for micro-RNA and protein detection, which are important biomarkers for cancer diagnosis [28]. The corresponding dye-labelled detection probes (FAM-ssDNA or FITC thrombin aptamer) were attached to the MoS_2_ surface via π–π interactions, resulting in rapid quenching of the fluorescent signal. Free navigation of the micromotors in solutions containing miRNA-21 or thrombin targets results in the release of the labelled probe and recovery of the fluorescent signal (see microscopy images in the figure).

## 4. In Vivo Biosensing

**Figure 7:**
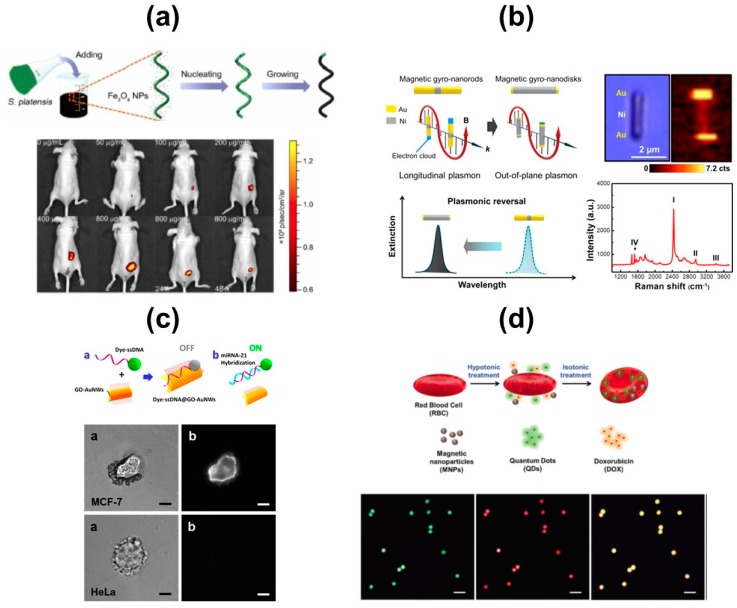
In vivo biosensing using self-propelled micromotors. (**a**) Spiruline-based magnetite micromotors for bioimaging, schematic of the preparation and operation. (**b**) Plasmonic-magnetic gyro-nanodisks for SERS bioassays. (**c**) microRNA detection using ultrasound propelled nanowire motors, schematic of the operation and detection in different cancer cell lines. (**d**) Red blood cell micromotors with quantum dots for bioimaging. Reproduced with permission from ref. [30] (**a**), The American Association for the Advancement of Science; ref. [31], (**b**) American Chemical Society; ref. [33] (**c**) from American Chemical Society and ref. [34] (**d**), Royal Society of Chemistry.

Compared with previous approaches using catalytic micromotors for biosensing, progress in this direction is still in early stages. Yet, promising proof-of-concept applications have been demonstrated so far. All of them rely on nano- and micromotors propelled by external stimuli, since peroxide fuel can be toxic to biological cells. Figure 7a,b shows approaches based on magnetic-propelled micromotors for in vivo biosensing approaches. In the first one, spiruline microalgae was used as a template for dip-coating of magnetic Fe_3_O_4_ microparticles, which impart them with magnetic properties, exhibiting negligible toxicity at the same time. In addition, the native microalgae fluorescence enables in vivo fluorescence imaging and remote diagnostic biosensing without the need for any surface modification [30]. The proof-of-concept was probed for influenza virus (HA1) detection. Magnetic gyronanodisks (GNDs) have been used for Fourier transform surface plasmon resonance-based biodetection. After incorporating the specific antibody to the surface, the disks are able to interact with the analyte, increasing the hydrodynamic force which causes a perturbation in the dynamics of the GNDs, which can be analyzed by observing changes in peak frequencies with regards to the target concentrations (see Figure 6b) [31].

Ultrasound propulsion is also a convenient approach for in vivo biosensing schemes. Early studies from Wang’s group demonstrated that antibody-functionalized, ultrasound-propelled Au–-Ni–Au nanowires can be used for bacteria isolation [32]. A most sophisticated strategy for micro-RNA sensing is depicted in Figure 7c. Dye-labelled single-stranded-DNA/graphene oxide-coated Au nanowires are first internalized into the cell. Before internalization, the fluorescence of the dye is quenched due to its attachment to the graphene oxide. Once in the cell, fluorescence is recovered due to the displacement of the dye–DNA probe upon binding with the target miRNA, allowing precise and real-time monitoring of intracellular miRNA expression [33]. To increase biocompatibility, iron oxide nanoparticles and CdTe quantum dots have been encapsulated in red blood cells (see Figure 4d), holding considerable promise for theragnostic and in vivo biosensing approaches [34]. 

## 5. Conclusions and Future Directions

This review has given a comprehensive overview of the current challenges and future prospects on the use of micromotors for biosensing applications. We have described first the different designs, synthetic strategies and propulsion mechanisms used in the fabrication of the micromotor body, which plays an important role in the subsequent biofunctionalization and final application. Catalytic micromotors based on bubble-propulsion are important for in vitro detection schemes. Magnetic and ultrasound-propelled micromotors are ideal for in vivo detection schemes (see Table 2).

The emerging field of micromotors is successfully adding a novel and rich dimension to the biosensor field: low-cost, simplification, and true miniaturization. Research efforts and new developments in the future should be aimed at the implementation of in vivo detection schemes, increasing the compatibility and functionality of the existing micromotors. The promising results in this direction, and the efficient coupling of micromotors with tomography and magnetic resonance equipment (commonly used in diagnosis) will probably lead to new developments in the near future. In connection with in vitro biosensing approaches based on micromotors, such developments will open new avenues in analytical chemistry, biosensing and even personalized medicine.

## Figures and Tables

**Table 1:** Micromotors for biosensing applications.

Micromotor	Biosensing Element	Detection Mechanism	Analyte	LB	Ref.
	**Catalytic propulsion**				
Au-Pt nanowires	Oligonucleotides	Motion based	DNA RNA	Low	[15]
Au-PPy nanowires	Glucose oxidase Glutamate oxidase Xantina oxidase	Motion based	Glucose Glutamate Xantine	Low	[16]
PEDOT-Au micromotors	DNA-Pt NPs	Motion based	DNA	Low	[17,18]
Ti/Fe/Au/Pt rolled-up micromotors	Antibody	Optical	Hela cancer cells	Low	[19]
AuNPs-PANI/Pt tubular micromotors	Antibody	Optical	Proteins	Low	[20]
PABA/Ni/Pt tubular micromotors	Built-in	Optical	Yeast cells	Low	[21]
MIP-PEDOT/Pt tubular micromotors	Built-in	Fluorescent	Proteins (avidin-FTIC)	Low	[22]
PEDOT/Ni/Pt tubular micromotors	Antibody	Colorimetric	Cortisol	Low	[23]
MnO_2_/Ni/Au nanosheets	Aptamer	Electrochemical	HL-60 cancer cells	Low	[24]
PCL-PtNPs Janus micromotors	PABA functionalized GQDS	Fluorescent	Endotoxins	Low	[25,26]
Graphene/Pt	Aptamers	Fluorescent	Toxins (ricin)	Low	[27]
MoS_2_/Pt	Dye-labeled DNA Aptamers	Fluorescent	DNA Thrombin	Low	[28]
	**Magnetic propulsion**				
PNIPAM-co-ABP-AAc/Ti/Fe rolled-up microtubes	-	Optical	Sperm cells	High	[29]
Microalgae/Fe_3_O_4_ helices	Native algae fluorescent	Optical MRI	Bioanalytes In vivo imaging	High	[30]
Au-Ni-Au nanowires	Antibody	SERS	Influenza virus	High	[31]
	**Ultrasound propulsion**				
Au-Ni-Au nanowires	Antibody	Optical	*Escherichia Coli* *Staphylococcus Aureus*	High	[32]
Au-graphene nanowires	Dye-labeled single-stranded DNA	Fluorescent	microRNA	High	[33]
Red blood cell-Fe_3_O_4_ NPs	CdTe quantum dots	Fluorescent	-	High	[34]

Note: LB: level of biocompatibility; PPy: polypyrrole; PEDOT: Poly(3,4-ethylenedioxythiophene); PANI: polyaniline; NPs: nanoparticles; PABA: poly (3-aminophenylboronic acid); PCL: polycaprolactone; PNIPAM-co-ABP-AAc poly(*N*-isopropylacrylamide)-co-acryloylbenzophenone-co-(acrylic acid).

**Table 2:** Advantages and disadvantages of nano- and micromotors for biosensing applications.

Propulsion	In Vivo Detection	In Vitro Detection
**Catalytic**	Low biocompatibility Negligible applicability Requires extremely low peroxide levels Enzyme motors: hampered locomotion in salt-rich environments	Easy functionalization Enhanced mixing Improved kinetics High towing force High versatility Practical applicability
**Magnetic**	High biocompatibility Do not require fuel Easy targeted delivery Easy functionalization	Easy functionalization Low reaction kinetics Limited applicability
**Ultrasound**	High biocompatibility Do not require fuel Can easily diffuse into cells	Easy functionalization Low reaction kinetics Limited applicability

## References

[1] Clark L.C., Lyons C. (1962). Electrode systems for continuous monitoring in cardiovascular surgery. Ann. N. Y. Acad. Sci..

[2] Gooding J.J. (2006). Nanoscale biosensors: Significant advantages over larger devices?. Small.

[3] Soleymani L., Li F. (2017). Mechanistic challenges and advantages of biosensor miniaturization into the nanoscale. ACS Sens..

[4] Paxton W.F., Kistler K.C., Olmeda C.C., Sen A., St Angelo S.K., Cao Y., Mallouk T.E., Lammert P.E., Crespi V.H. (2004). Catalytic nanomotors: Autonomous movement of striped nanorods. J. Am. Chem. Soc..

[5] Ozin G.A., Manners I., Fournier-Bidoz S., Arsenault A. (2005). Dream nanomachines. Adv. Mater..

[6] Pantarotto D., Browne W.R., Feringa B.L. (2008). Autonomous propulsion of carbon nanotubes powered by a multienzyme ensemble. Chem. Commun..

[7] Gao W., Sattayasamitsathit S., Orozco J., Wang J. (2011). Highly efficient catalytic microengines: Template electrosynthesis of polyaniline/platinum microtubes. J. Am. Chem. Soc..

[8] Mei Y., Solovev A.A., Sanchez S., Schmidt O.G. (2011). Rolled-up nanotech on polymers: From basic perception to self-propelled catalytic microengines. Chem. Soc. Rev..

[9] Wang J. (2013). Nanomachines: Fundamentals and Applications.

[10] Karshalev E., Esteban-Fernandez de Avila B., Wang J. (2018). Micromotors for “Chemistry-on-the-Fly”. J. Am. Chem. Soc..

[11] Campuzano S., Esteban-Fernandez de Avila B., Yanez-Sedeño P., Pingarron J.M., Wang J. (2017). Nano/microvehicles for efficient delivery and (bio)sensing at the cellular level. Chem. Sci..

[12] Kim K., Guo J., Liang Z., Fan D. (2018). Artificial micro/nanomachines for bioapplications: Biochemical delivery and diagnostic sensing. Adv. Funct. Mater..

[13] Wang J. (2016). Self-propelled affinity biosensors: Moving the receptor around the sample. Biosens. Bioelectron..

[14] Gao W., de Ávila B.E.-F., Zhang L., Wang J. (2018). Targeting and isolation of cancer cells using micro/nanomotors. Adv. Drug Deliv. Rev..

[15] Wu J., Balasubramanian S., Kagan D., Manesh K.M., Campuzano S., Wang J. (2010). Motion-based DNA detection using catalytic nanomotors. Nat. Commun..

[16] Bunea A.I., Pavel I.A., David S., Gaspar S. (2015). Sensing based on the motion of enzyme-modified nanorods. Biosens. Bioelectron..

[17] Van Nguyen K., Minteer S.D. (2015). DNA-functionalized Pt nanoparticles as catalysts for chemically powered micromotors: Toward signal-on motion-based DNA biosensor. Chem. Commun..

[18] Fu S., Zhang X., Xie Y., Wu J., Ju H. (2017). An efficient enzyme-powered micromotor device fabricated by cyclic alternate hybridization assembly for DNA detection. Nanoscale.

[19] Balasubramanian S., Kagan D., Hu C.J., Campuzano S., Lobo-Castañon M.J., Lim N., Kang D.Y., Zimmerman M., Zhang L., Wang J. (2011). Micromachine-enabled capture and isolation of cancer cells in complex media. Angew. Chem. Int. Ed..

[20] Yu X., Li Y., Wu J., Ju H. (2014). Motor-based autonomous microsensor for motion and counting immunoassay of cancer biomarker. Anal. Chem..

[21] Kuralay F., Sattayasamitsathit S., Gao W., Uygun A., Katzenberg A., Wang J. (2012). Self-propelled carbohydrate-sensitive microtransporters with built-in boronic acid recognition for isolating sugars and cells. J. Am. Chem. Soc..

[22] Orozco J., Cortés A., Cheng G., Sattayasamitsathit S., Gao W., Feng X., Shen Y., Wang J. (2013). Molecularly imprinted polymer-based catalytic micromotors for selective protein transport. J. Am. Chem. Soc..

[23] Esteban-Fernandez de Avila B., Zhao M., Campuzano S., Ricci F., Pingarron J.M., Mascini M., Wang J. (2017). Rapid micromotor-based naked-eye immunoassay. Talanta.

[24] Amouzadeh Tabrizi M., Shamsipur M., Saber R., Sarkar S. (2018). Isolation of HL-60 cancer cells from the human serum sample using MnO_2_-PEI/Ni/Au/aptamer as a novel nanomotor and electrochemical determination of thereof by aptamer/gold nanoparticles-poly(3,4-ethylene dioxythiophene) modified GC electrode. Biosens. Bioelectron..

[25] Jurado-Sánchez B., Pacheco M., Rojo J., Escarpa A. (2017). Magnetocatalytic graphene quantum dots Janus micromotors for bacterial endotoxin detection. Angew. Chem. Int. Ed..

[26] Pacheco M., Jurado-Sánchez B., Escarpa A. (2018). Sensitive monitoring of enterobacterial contamination of food using self-propelled Janus microsensors. Anal. Chem..

[27] Esteban-Fernández de Ávila B., Lopez-Ramirez M.A., Báez D.F., Jodra A., Singh V.V., Kaufmann K., Wang J. (2016). Aptamer-modified graphene-based catalytic micromotors: Off–on fluorescent detection of ricin. ACS Sens..

[28] Singh V.V., Kauffman K., Esteban-Fernández de Ávila B., Karshalev E., Wang J. (2016). Molybdenum disulfide-based tubular microengines: Toward biomedical applications. Adv. Funct. Mater..

[29] Magdanz V., Guix M., Hebenstreit F., Schmidt O.G. (2016). Dynamic polymeric microtubes for the remote-controlled capture, guidance, and release of sperm cells. Adv. Mater..

[30] Yan X., Zhou Q., Vincent M., Deng Y., Yu J., Xu J., Xu T., Tang T., Bian L., Wang Y.-X.J. (2017). Multifunctional biohybrid magnetite microrobots for imaging-guided therapy. Sci. Robot..

[31] Jung I., Ih S., Yoo H., Hong S., Park S. (2018). Fourier transform surface plasmon resonance of nanodisks embedded in magnetic nanorods. Nano Lett..

[32] Garcia-Gradilla V., Orozco J., Sattayasamitsathit S., Soto F., Kuralay F., Pourazary A., Katzenberg A., Gao W., Shen Y., Wang J. (2013). Functionalized ultrasound-propelled magnetically guided nanomotors: Toward practical biomedical applications. ACS Nano.

[33] Esteban-Fernández de Ávila B., Martín A., Soto F., Lopez-Ramirez M.A., Campuzano S., Vásquez-Machado G.M., Gao W., Zhang L., Wang J. (2015). Single cell real-time miRNAs sensing based on nanomotors. ACS Nano.

[34] Wu Z., Esteban-Fernandez de Avila B., Martin A., Christianson C., Gao W., Thamphiwatana S.K., Escarpa A., He Q., Zhang L., Wang J. (2015). RBC micromotors carrying multiple cargos towards potential theranostic applications. Nanoscale.

[35] Campuzano S., Orozco J., Kagan D., Guix M., Gao W., Sattayasamitsathit S., Claussen J.C., Merkoçi A., Wang J. (2012). Bacterial isolation by lectin-modified microengines. Nano Lett..

[36] Medina-Sánchez M., Schwarz L., Meyer A.K., Hebenstreit F., Schmidt O.G. (2016). Cellular cargo delivery: Toward assisted fertilization by sperm-carrying micromotors. Nano Lett..

[37] Kline T.R., Paxton W.H., Mallouk T.E., Sen A. (2005). Catalytic nanomotors: Remote-controlled autonomous movement of striped metallic nanorods. Angew. Chem. Int. Ed..

[38] Wang W., Duan W., Ahmed S., Mallouk T.E., Sen A. (2013). Small power: Autonomous nano- and micromotors propelled by self-generated gradients. Nano Today.

[39] Wang J. (2014). Template electrodeposition of catalytic nanomotors. Faraday Discuss..

[40] Maria-Hormigos R., Jurado-Sanchez B., Vazquez L., Escarpa A. (2016). Carbon allotrope nanomaterials based catalytic micromotors. Chem. Mater..

[41] Solovev A.A., Mei Y., Bermúdez Ureña E., Huang G., Schmidt O.G. (2009). Catalytic microtubular jet engines self-propelled by accumulated gas bubbles. Small.

[42] Jurado-Sánchez B., Pacheco M., Maria-Hormigos R., Escarpa A. (2017). Perspectives on Janus micromotors: Materials and applications. Appl. Mater. Today.

[43] Dey K.K., Zhao X., Tansi B.M., Méndez-Ortiz W.J., Córdova-Figueroa U.M., Golestanian R., Sen A. (2015). Micromotors powered by enzyme catalysis. Nano Lett..

[44] Ma X., Hortelão A.C., Patiño T., Sánchez S. (2016). Enzyme catalysis to power Micro/Nanomachines. ACS Nano.

[45] Patiño T., Feiner-Gracia N., Arqué X., Miguel-López A., Jannasch A., Stumpp T., Schäffer E., Albertazzi L., Sánchez S. (2018). Influence of enzyme quantity and distribution on the self-propulsion of non-Janus urease-powered micromotors. J. Am. Chem. Soc..

[46] Nicewarner-Peña S.R., Freeman R.G., Reiss B.D., He L., Peña D.J., Walton I.D., Cromer R., Keating C.D., Natan M.J. (2001). Submicrometer metallic barcodes. Science.

[47] Wang H., Pumera M. (2015). Fabrication of micro/nanoscale motors. Chem. Rev..

[48] Gao W., Kagan D., Pak O.S., Clawson C., Campuzano S., Chuluun-Erdene E., Shipton E., Fullerton E.E., Zhang L., Lauga E. (2012). Cargo-towing fuel-free magnetic nanoswimmers for targeted drug delivery. Small.

[49] Laocharoensuk R., Burdick J., Wang J. (2008). Carbon-nanotube-induced acceleration of catalytic nanomotors. ACS Nano.

[50] Luis B.P., Jahir O., Pablo G., Arben M. (2018). Architecting Graphene oxide rolled-up micromotors: A simple paper-based manufacturing technology. Small.

[51] Jurado-Sánchez B., Escarpa A. (2016). Milli, micro and nanomotors: Novel analytical tools for real-world applications. Trends Anal. Chem..

[52] Maria-Hormigos R., Jurado-Sanchez B., Escarpa A. (2017). Tailored magnetic carbon allotrope catalytic micromotors for ‘on-chip’ operations. Nanoscale.

[53] Gao W., Feng X., Pei A., Kane C.R., Tam R., Hennessy C., Wang J. (2014). Bioinspired helical microswimmers based on vascular plants. Nano Lett..

[54] Li T., Li J., Zhang H., Chang X., Song W., Hu Y., Shao G., Sandraz E., Zhang G., Li L. (2016). Magnetically propelled fish-like nanoswimmers. Small.

[55] Srivastava S.K., Medina-Sanchez M., Koch B., Schmidt O.G. (2016). Medibots: Dual-action biogenic microdaggers for single-cell surgery and drug release. Adv. Mater..

[56] Chen X.-Z., Hoop M., Mushtaq F., Siringil E., Hu C., Nelson B.J., Pané S. (2017). Recent developments in magnetically driven micro- and nanorobots. Appl. Mater. Today.

[57] Tu Y., Peng F., Wilson D.A. (2017). Motion manipulation of micro- and nanomotors. Adv. Mater..

[58] Wu Z., Li T., Li J., Gao W., Xu T., Christianson C., Gao W., Galarnyk M., He Q., Zhang L. (2014). Turning erythrocytes into functional micromotors. ACS Nano.

[59] Wang W., Castro L.A., Hoyos M., Mallouk T.E. (2012). Autonomous motion of metallic microrods propelled by ultrasound. ACS Nano.

[60] Xu T., Xu L.-P., Zhang X. (2017). Ultrasound propulsion of micro-/nanomotors. Appl. Mater. Today.

[61] Moo J.G.S., Pumera M. (2016). Self-propelled micromotors monitored by particle-electrode impact voltammetry. ACS Sens..

[62] Vilela D., Cossio U., Parmar J., Martinez-Villacorta A.M., Gomez-Vallejo V., Llop J., Sanchez S. (2018). Medical imaging for the tracking of micromotors. ACS Nano.

[63] Orozco J., Jurado-Sánchez B., Wagner G., Gao W., Vazquez-Duhalt R., Sattayasamitsathit S., Galarnyk M., Cortés A., Saintillan D., Wang J. (2014). Bubble-propelled micromotors for enhanced transport of passive tracers. Langmuir.

[64] Kagan D., Calvo-Marzal P., Balasubramanian S., Sattayasamitsathit S., Manesh K.M., Flechsig G.-U., Wang J. (2009). Chemical sensing based on catalytic nanomotors: Motion-based detection of trace silver. J. Am. Chem. Soc..

[65] Orozco J., Pan G., Sattayasamitsathit S., Galarnyk M., Wang J. (2015). Micromotors to capture and destroy anthrax simulant spores. Analyst.

